# Study protocol of the YP Face IT feasibility study: comparing an online psychosocial intervention versus treatment as usual for adolescents distressed by appearance-altering conditions/injuries

**DOI:** 10.1136/bmjopen-2016-012423

**Published:** 2016-10-03

**Authors:** Heidi Williamson, Claire Hamlet, Paul White, Elsa M R Marques, Julia Cadogan, Rohan Perera, Nichola Rumsey, Leighton Hayward, Diana Harcourt

**Affiliations:** 1 Faculty of Health and Applied Sciences, Centre for Appearance Research, University of the West of England Bristol, Bristol, UK; 2 Centre for Appearance Research, University of the West of England, Bristol, UK; 3 University of the West of England Bristol, Bristol, UK; 4 School of Social and Community Medicine, University of Bristol and the National Institute for Health Research (NIHR) Collaboration for Leadership in Applied Health Research, Bristol, UK; 5 University Hospitals Bristol NHS Foundation Trust, Bristol, UK; 6 Pembroke Road Surgery, Bristol Clinical Commissioning Group, Bristol, UK; 7 PPI Representative, Lincoln, UK

**Keywords:** Adolescents, Appearance-related distress, Cognitive Behavioural Therapy, Online intervention, Disfigurement, Visible Difference

## Abstract

**Introduction:**

A significant number of adolescents suffer extensive and enduring difficulties such as social anxiety, body image dissatisfaction, low self-esteem and bullying as a result of conditions or injuries that affect their appearance (eg, craniofacial and skin conditions, treatment side effects and scarring). Evidence-based psychosocial interventions to meet their specific needs are currently lacking. YP Face IT, developed by the UK's Centre for Appearance Research in collaboration with clinical experts and young people, is an innovative online psychosocial intervention designed to offer this group immediate support, advice and coping strategies. It has been endorsed by young people, their parents/carers, GPs, clinical psychologists and health professionals working with those affected by appearance-related conditions.

**Methods and analysis:**

Young people aged 12–17 with an appearance-altering condition/injury that self-identify as experiencing appearance-related distress, teasing or bullying will be invited to participate via GP practices and UK charities. Consenting participants will be randomised to the intervention (YP Face IT) or the treatment as usual (TAU) control group. Outcome measures will be completed by young people and their parents/carers at baseline, 13, 26 and 52 weeks. Primary outcome measures will be the Body Esteem Scale and the Social Anxiety Scale for Adolescents. Participants will complete other health-related outcome measures and resource use questionnaires for health economic analysis. We will assess recruitment rates, acceptability of the YP Face IT programme, adherence and retention to treatment, questionnaire completion rates, variation of TAU in Primary Care and the feasibility of GP practice staff supervising young people's use of YP Face IT.

**Ethics and dissemination:**

This feasibility trial protocol (V.1, 3 March 2014), received a favourable ethical opinion from the NRES Committee South West-Frenchay (reference number 14/SW/0058). Findings will be disseminated through academic peer-reviewed publications, conferences and to participating GP practices and charities supporting those with conditions affecting appearance.

**Trial registration number:**

ISRCTN40650639; Pre-results.

**Strengths and limitations of this study**

- This is the first feasibility study to evaluate an online psychosocial intervention for young people living with a visible difference in an NHS Primary Care setting.
- Public and patient involvement has informed the intervention and study design.
- Feasibility data will be supplemented by qualitative data from young people, their parents/carers and GP practice staff.
- Participant outcomes, including 12-month follow-up, are self-reported.
- Participants are not blinded to allocation and this could lead to reporting bias.

## Introduction

Looking ‘different’ due to disease, treatments, injury or congenital conditions (eg, skin conditions, scarring, cleft lip and palate) can have a significant psychosocial impact during adolescence.^1–4^ While it is important to highlight that many people living with a visible difference (‘disfigurement’) adjust well to their condition and report increased well-being, self-acceptance and stronger social relationships,^5^ around a third of young people living with a visible difference suffer extensive and enduring difficulties, including bullying, low self-esteem, body image dissatisfaction, social anxiety and avoidance, poor quality of life and academic performance.^6–9^ Anxiety and avoidance interfere with social and emotional development and, if not addressed, can become chronic and disabling, leading to anxiety disorders which are an economic burden for society.^10^

Research consistently shows that location, size and cause of a visible difference do not accurately predict levels of appearance-related distress.^11^ ^12^ Rather, well-being is often determined by intervening sociocognitive factors, including social confidence, perceptions of social acceptance and fear of negative evaluation.^13^ These factors are amenable to change via psychosocial interventions that encourage the development of self-management skills and offer an adjunct or alternative to medical and surgical solutions.^14^

### Psychosocial interventions for appearance-related distress

Evidence-based psychosocial interventions to meet the specific needs of young people with appearance-related distress associated with a visible difference are currently lacking.^15^ ^16^ There is some evidence, albeit limited, that interventions based on cognitive–behaviour therapy (CBT) and social interaction skills training (SIST) may be as effective for young people^15^ as evidence suggests they are for adults.^14^ ^17^ Face IT is a successful online intervention for adults that uses SIST and CBT for appearance-related distress. Its therapeutic content was informed by Kent's^18^ Model of Psychosocial Distress and Intervention for Individuals with Visible Differences which purports that an unusual appearance, perceived as unattractive by the individual, can increase negative appearance-related cognitions and fear of rejection. When combined with experiences of social stigma, these experiences can heighten social anxiety, resulting in individuals appearing distracted or anxious, which inhibits effective social functioning.^19^ In a randomised controlled trial (RCT), Face IT reduced anxiety-related concerns and was comparable to a face-to-face CBT intervention. These improvements increased at 6-month follow-up.^20^

### Developing young person's Face IT (YP Face IT)

The project to develop and evaluate a young persons' version of Face IT is following the Medical Research Council framework for the development of complex interventions.^21^ To date, this has involved a series of acceptability studies with young people with a range of appearance-altering conditions (including those with skin/craniofacial conditions and scarring), their parents, clinical experts (psychologists, consultants and specialist nurses working with those affected by appearance-altering conditions) and General Practitioners (GPs).^16^ In these studies, participants rejected adult Face IT as a suitable intervention and collaborated with researchers to develop a youth version (YP Face IT), with similar therapeutic content but revamped to meet the specific developmental and cultural needs of adolescents. YP Face IT (http://www.ypfaceit.co.uk) has attractive multimedia presentations and is appropriate for 12–17 years with a reading age of 12 years and readability levels of 90–100%.^22^

Young people have endorsed YP Face IT over face-to-face therapy because it can be accessed in private, at their convenience and because they would feel less embarrassed/inhibited taking part.^16^ Psychologists have welcomed its potential to address a current gap in care provision and to overcome barriers that prevent young people and parents using their services, including needing time off school/work, travelling, perceived stigma of therapy^23^ and social anxiety/avoidance: a defining characteristic of this group.^24^ GPs, parents and young people have also requested that YP Face IT be made widely available with minimal limitations on who can access it, as GPs report they struggle to identify appropriate evidence-based appearance-specific psychological interventions.^25^ In addition, those with appearance-related distress rarely meet criteria for referral to Child and Adolescent Mental Health Services (CAMHS) and many receiving secondary care for their condition have no, or limited, access to a psychologist.^16^ Providing easily accessible interventions is in line with current policy^26^ and calls for innovations to improve access to primary care services.^27^

YP Face IT is an innovative, widely acceptable intervention offering immediate support, advice and strategies to young people affected by appearance-related distress, with potential benefits in terms of improved quality of life for a large and diverse user group. Advantages centre around addressing an unmet need and targeting low body image and social anxiety; which have multiple short-term and long-term impacts on public health and NHS resource use.^4^ ^28–30^

## Methods and analysis

### Participants and recruitment

The target population is 12–17 years living with a visible difference who self-identify as experiencing appearance-related distress, teasing or bullying. Charities supporting those with a visible difference (eg, http://www.changingfaces.org.uk) will be approached to ask them to advertise the study via their websites and newsletters to young people and parents. The main avenue for recruitment will be through primary care general practices that do not specialise in appearance-related issues; sites in Bristol and surrounding areas in the South West of the UK will be invited to take part. To increase representativeness of participants from a range of socioeconomic backgrounds, we will aim to include practices with a range of index of multiple deprivation scores.

Based on advice from our YP Face IT advisory group (including GPs, parents/carers and young people), patients from GP practices will be made aware of the study in three ways; through targeted letters of invitation and, to enable introduction of the study to those whose appearance-altering condition is not recorded in their medical notes, via posters/leaflets displayed in the surgery and during consultations. How participants learnt about the study will be recorded to inform recruitment for a future RCT.

With the support of the West of England NIHR Clinical Research Network, a comprehensive list of conditions/injuries known to impact appearance were used to create a specific list of READ codes (medical diagnosis coding system used by practices). GP practices recruited to take part in the study will use these codes to identify eligible patients. GPs will review this list and at their discretion remove those deemed not suitable for invitation (eg, those whose condition has resolved). Young people will be posted an information pack, including a GP invitation letter and information sheet. For those under 16 years, letters will be addressed to their parents/carers who will be asked to discuss participation with their child. A reminder will be sent 4 weeks later to non-respondents, this will include a response form for indicating why they decided not to take part and self-addressed envelope.

Doctors and nurses from GP practices will be asked to recruit young people who meet the inclusion criteria during consultations. We anticipate that the GP practices we recruit will not have extensive experience of raising the topic of appearance with young people; therefore, GPs and nurses will receive training from the YP Face IT team on how to approach and sensitively introduce the study. Staff will provide interested young people with information sheets and leaflets. These will include details for contacting the research team. Posters, designed with input from the advisory group, will be displayed in participating practices inviting young people to contact their GP if the study is of relevance.

### Inclusion criteria

Young people aged 12–17 years with any appearance-altering condition, injury or treatment side effect, who self-identify as experiencing appearance-related distress, teasing or bullying and their parent/carer will be eligible. The cause, location and severity of the visible difference will not be objectively assessed, as research consistently finds physical characteristics do not relate to distress levels.^11^ Participants under 16 years will require a parent/carer to join the study, those aged 16 or above will be encouraged to inform and involve their parent/carer, but this is not mandatory. Participants need to be fluent in English (audio clips are built in to the programme for those who struggle reading text) with normal/corrected-to-normal vision and access to a private computer or tablet.

### Exclusion criteria

We will exclude participants with a learning disability that compromises their ability to provide informed consent. Those currently receiving a psychological intervention, diagnosed with clinical depression, psychosis, eating disorder or posttraumatic stress disorder (PTSD) and those within 12 months of a traumatic injury will be excluded. PTSD is a risk for those disfigured through trauma.^31^ GP practices will be asked to screen for study eligibility before inviting young people to participate. Confirmation of this eligibility will rely on parent and young people's self-report when the researcher speaks to them over the telephone before informed consent is obtained. An example of the consent form can be found in online supplementary appendix 1.

10.1136/bmjopen-2016-012423.supp1 supplementary appendix

### Study design

The study will be a 2-year feasibility RCT starting July 2014. Remote randomisation will be provided by the Bristol Randomised Trials Collaborative based at the University of Bristol, with eligible participants randomised to ‘Treatment as Usual’ (TAU-control group) or YP Face IT in addition to TAU (intervention group). Block randomisation will help ensure equal numbers in each practice are allocated to each condition. A pragmatic parallel-groups RCT, using current practice as the control arm, was acceptable to our advisory group and is the most methodologically robust design to measure self-report psychological outcomes. Randomisation will ensure that in the definitive trial, effects due to condition progression will be equally distributed across groups. Participants who meet our inclusion criteria and their parent/carer will be consented by the research team and asked to provide demographic information. At baseline, young people and their parent/carer will be asked to complete their outcome measures, consented participants will then be randomised to the TAU-control or intervention group. Owing to the nature of the intervention, neither the participants, GP practice staff nor researchers can be blinded to allocation. Participants will be informed that this is a feasibility study and that YP Face IT has not yet been proven effective. Online supplementary appendix 2 details the feasibility trial process diagrammatically.

10.1136/bmjopen-2016-012423.supp2 supplementary appendix

### Treatment as usual

Participants in the control arm will continue with TAU and complete their outcome measures at baseline and 13, 26 and 52-week follow-up. Feedback from GP advisors confirms TAU varies widely, including limited advice, referral to Mood Gym^32^ (an online intervention ie, not appearance-specific), inpractice counsellors or onward referral to CAMHS. This is a pragmatic trial and no participants will be denied access to alternative treatments and services offered by the NHS or other organisations. Details of the type and frequency of TAU received will be collected via health economic data collection tools, note reviews and interviews.

### Intervention

YP Face IT will be provided in addition to TAU for those randomised to the intervention group. Participants will also complete outcome measures at baseline and 13, 26 and 52-week follow-up. The intervention consists of 7 weekly sessions each lasting around 30–40 min, and a booster session 6 weeks later to maintain therapeutic effect. Sessions provide advice and support in written, audio and video formats and include interactive and homework activities that teach and encourage users to practise strategies such as managing staring or bullying. Social skills, anxiety-management and CBT techniques are taught to overcome social anxiety and target negative appearance-related thoughts and behaviours that reduce self-esteem. Video inserts feature adolescent actors with a visible difference, who play different roles in scenarios scripted and created for the programme. Participants can hear testimonies from celebrity role models with appearance-altering conditions, learn from others who have adjusted positively to the challenges of living with a visible difference and can reflect on their own experiences via an online diary. To facilitate adherence to the intervention, participants identify a suitable day and time to complete their next session using the YP Face IT calendar. They (and a parent/carer if they choose) receive automated emails and/or texts 24 and 1 hour before to remind them to complete the session. The content of YP Face IT is organised as follows:

- Session 1: Common problems
- Session 2: Improve your social skills
- Session 3: Don't be SCARED, REACH OUT
- Session 4: ‘Think, Feel, Do’
- Session 5: SMART goals
- Session 6: Beating anxiety
- Session 7: Looking at your progress
- Session 8: Booster quiz

Young people randomised into the intervention group will receive an email with the YP Face IT website link and a secure username and password to access the intervention. Participants complete the intervention at home or in a location of their choice using a computer/tablet. Participants' use of the intervention will be monitored by the research team who have privileged access to data stored on the website. An administration area within YP Face IT records the participant's diary entries and qualitative responses to reflective activities and homework tasks and records their engagement with the website (pages viewed and time spent on each activity). In addition, to evaluate the feasibility of primary care staff supervising young people completing YP Face IT, 5–8 practices will receive training to enable them to access data and monitor their own patients.

### Trial design characteristics

**Figure 1:**
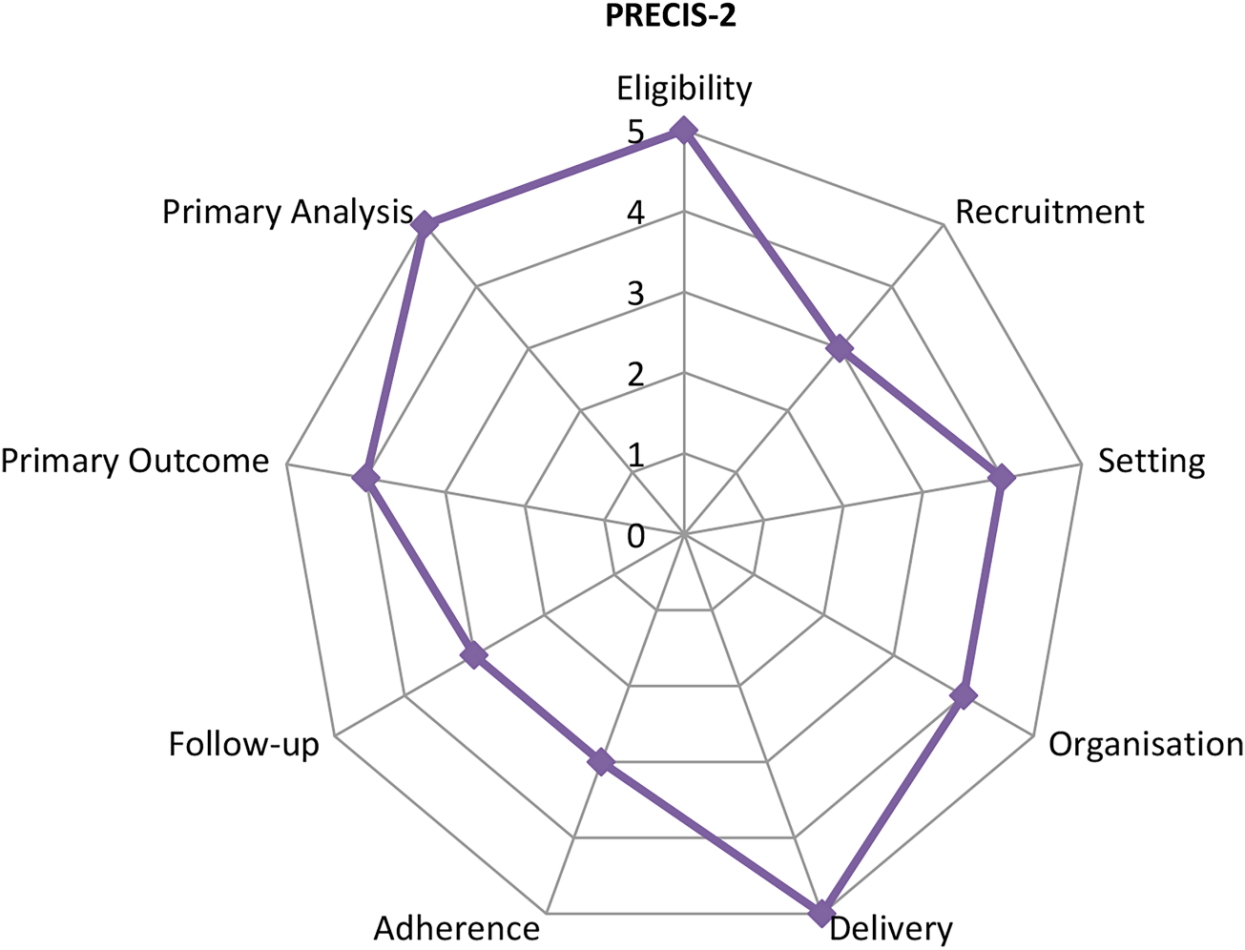
PRECIS-2.

We assessed the characteristics of the feasibility study using the pragmatic-explanatory continuum indicator summary (PRECIS-2)^33^ in relation to eligibility, recruitment, study settings, flexibility of intervention delivery and adherence, follow-up, primary outcome and primary analysis. PRECIS is an accepted tool for assessing trial design on a continuum of effectiveness versus efficacy. Pragmatic trials aim to evaluate the effectiveness of an intervention in a real-life setting and with a study population that is similar as possible to those that will use it. Exploratory trials aim to explore whether the intervention can work under ideal conditions, whereas a pragmatic approach aims to explore whether the intervention works under usual conditions.^33^ ^34^ Figure 1 shows the current feasibility study design based on the nine criteria. Specifically, eligibility, delivery and primary analysis follow more pragmatic approaches, while recruitment, setting, organisation, adherence follow-up and primary outcome fall along the pragmatic-explanatory continuum. We will consider amendments to the domains within the PRECIS tool when designing a full RCT in order, to improve the external validity of any definitive results.

### Feasibility outcome

The study aims to establish whether it is acceptable and feasible to conduct an RCT to evaluate YP Face IT as an adjunct to TAU in a primary care setting, with specific consideration of:

- Adherence and retention to treatment and outcome measures;
- Participants' experiences of using the intervention or being randomised to receive TAU only;
- The variation of TAU provided by GP practices;
- Participants' views on data collection processes and responses to self-report measures (including completion rates and time to complete) to determine the suitability of the outcome measures;
- Choice of, and ability to collect, data for a health economic evaluation;
- The feasibility of GP practice staff supervising young people's use of YP Face IT;
- The suitability of processes for ensuring participants are supported as they work through the intervention and receive onward referral, if necessary;
- Numbers of eligible participants recruited via GP practices.

### Clinical outcome

We will assess the impact of YP Face IT on various psychosocial outcome measures, completed by participants online using the Qualtrics data capture tool (http://www.qualtrics.com), which have been chosen based on their scientific merit and use in appearance-related research.

### Primary outcome measures

#### Body Esteem Scale

This self-report scale for adolescents and adults measures body esteem (BE)—a self-evaluation of one's body or appearance.^35^ The BE-appearance subscale (10 items) was selected for the current study as it refers to general feelings about appearance. Feelings are rated on a 5-point scale from 0 (never) to 4 (always).

The BE Scale was chosen over other measures such as the Multidimensional Body-Self Relations Questionnaire^36^ as it is validated with children and adolescents. Additionally, young people from our advisory group preferred it as it was quick and easy to complete.

#### Social Anxiety Scale for Adolescents scale

This self-report scale for adolescents assesses feelings of social anxiety in the context of peer relations.^37^ It contains 22 items and asks young people to describe how they feel in relation to each statement. Feelings are rated on a 5-point scale ranging from 1 (not at all) to 5 (all the time). The parent version has the same items but asks the parent to rate their child's feelings and behaviour. This measure was chosen as social anxiety and avoidance is a major outcome of poor adjustment to a visible difference and evidence from our systematic review^15^ recommends its use.

### Secondary outcome measures

#### Social skills improvement system

This self-report scale assists professionals in screening and classifying young people suspected of having significant social skills deficits (eg, with communication and cooperation); scales are completed by the child and the parent.^38^ Young people indicate how true a statement is about each social skill and problem behaviour using a 4-point scale of 0 (not true) to 3 (a lot true). Parents indicate the frequency in which the young person exhibits each social skill. This is rated on a 4-point scale from 0 (never) to 3 (almost always). This measure was selected as there are very few validated measures available that evaluate the social skills that YP Face IT aims to improve. Additionally, an alternative to the SSIS that is commonly used^39^ was rejected by our advisors as having inappropriate items for those with visible differences (eg, those relating to ‘normal’ facial expressions).

### Self-perception profile

This self-report measure captures how one defines the self and contains nine subscales, each with five items.^40^ Young people are asked to choose which of two statements they are most like, and then decide whether the selected statement is ‘Really True for Me’ or ‘Sort of True for Me’. Only the Romantic Appeal (YP Face IT addresses common romantic concerns) and Global Self-Worth subscales have been selected for the current study. The measure was chosen because romantic issues and concerns are highly relevant to this group^41^ and are addressed within YP Face IT.

*Perceived Stigmatisation Questionnaire*^6^ This self-report measure assesses the extent to which young people experience stigmatising social behaviour. It has three subscales: the absence of friendly behaviour, confused and staring behaviour and hostile behaviour by others. Young people are asked to rate how often people act in a certain way towards them on a 5-point scale ranging from 1 (never) to 5 (always). This measure was selected to establish whether the strategies included within YP Face IT will impact on young people's perceived stigmatisation, as young people regard this outcome as highly salient to their quality of life.^42^

### EQ-5D-5L

This self-report measure assesses health status in order to provide a simple, generic measure of health for clinical and economic evaluation of healthcare.^43^ It was selected as it is suitable for those aged 12 years and over. The EQ-5D-5L comprises five dimensions: mobility, self-care, usual activities, pain/discomfort and anxiety/depression. It includes a visual analogue scale which asks respondents to self-rate their health from 0 (the worst health you can imagine) to 100 (the best health you can imagine).

### Qualitative outcomes

After completing the intervention (or at 13 weeks for the control group), a purposeful sample of 15–20 young people and parents/carers will be invited to participate in semistructured interviews lasting 20–30 min. We will offer the option of telephone interviews and continue to interview until data saturation has been reached. Using interview guides (see online supplementary appendix 3) developed with input from the advisory group, we will explore views on the acceptability and feasibility of recruitment processes, randomisation, retention and outcome measures. Intervention group interviews will also investigate experiences of completing YP Face IT. After 12 months, interviews will be conducted with a range of 10–12 young people (from the intervention and control group) to explore their experiences of the full study, including all follow-ups. Interviews will be digitally recorded and transcribed verbatim, before template data analysis^44^ is conducted by the researcher who will keep a reflective journal throughout.

10.1136/bmjopen-2016-012423.supp3 supplementary appendix

Practice staff supervising young people completing the intervention will be invited for interview when all of their patients randomised to YP Face IT have finished the intervention. This will explore the feasibility of GP practice staff supervising young people completing the programme, and their views on recruitment processes, barriers and facilitators to recruitment and retention.

### Health economic data collection and analysis

We will prepare the data collection methods needed to undertake an economic evaluation in a definitive trial, from a patient and parent/carer perspective. In the future, we expect YP Face IT to follow a commercialisation pattern such as that used by ‘Beating the Blues’, an online intervention for depression^45^ which is currently offered in primary care for a yearly licence fee per practice. We will collect the resource use required by the GP practice to deliver and support the running of YP Face IT in trial records, including staff grades and time necessary, and use hourly wages, on-costs and indirect costs to value staff's time. We will liaise with companies providing similar computerised healthcare to estimate licence fee cost. Other resource use will be collected from baseline until 12-month follow-up by reviewing GP records and parent-completed resource use questionnaires. Participant questionnaires will be completed online at 13, 26 and 52-week follow-up by the YP or parent, and will include resource use required to deliver healthcare in the community, use of social services, private expenses (eg, make up, wigs) and productivity losses, including parent/carer time off work and children's days off school. We will further cross check patient-reported community healthcare resource use by asking participating GP practices to review young people's GP records at 52 weeks. Resources will be valued using unit costs for health and social care,^46^ ^47^ the British National Formulary,^48^ ONS average weekly earnings,^49^ the AA (travel costs) or local sources (eg, computerised healthcare companies). We will employ sensitivity analysis to account for uncertainty in our costing assumptions (eg, cost of delivering the intervention). We will examine distributions and trends in costs and quality-adjusted life years to determine potential cost drivers and reduce sources of missing data in the future trial. If results from this study indicate a longer time frame is required to capture all health benefits and costs from the intervention, then we will plan for a decision analytical model in the future economic evaluation.

### Proposed sample size and data analysis

Quantitative data will be collected throughout the feasibility study to better understand the number of eligible young people, estimate willingness of young people and parents/carers to participate and the proportion finding randomisation acceptable. No formal power calculations are undertaken in feasibility studies; instead, a suitable number of participants are recruited to gain knowledge about factors such as attrition and recruitment in relation to feasibility outcomes.^50^ Using 10 sites, it is estimated a minimum of 100 young people would be eligible, with more than 60 providing consent for participation. These projected feasibility sample sizes allow consent percentage to be estimated with 95% confidence to within ±11%. Under a worst-case scenario, the projected feasibility sample sizes would allow acceptability rates and completion rates to be estimated with error margins of ±13%. Low participation in the TAU group would be a concern and a sample of 30 allocated to this group would have in excess of 80% power for detecting a 50% or lower participation rate against an anticipated rate 75% or higher and would therefore confidently discriminate between low and good participation rates.

Percentage missing values will be determined at each data collection stage and used to inform acceptability of the chosen outcome measures. Estimated participation rate per practice patient population and questionnaire completion percentages will be used to project the number of practices and sampling duration necessary for the sample size in an RCT. Simulation using participation rate information and response completion profiles of participants will inform these calculations. A feasibility sample size of 60 will ensure variation in individual questionnaire completion will be factored into the projected sample size determination. Findings will determine sensitivity to change of the proposed outcome measures and establish confidence in intervention effect. For a medium effect (Cohen's d=0.5), sample mean values for outcome measures based on 30 per group should align in the hypothesised direction of patient benefit ∼98% of the time. Sample sizes will permit SD of outcomes to be estimated within 25% of the true SD.

### Trial status

Recruitment of participants is completed. The first participant was enrolled in September 2014. The last participant will complete follow-up in September 2016.

## Ethics and dissemination

### Ethical and safety considerations

#### Obtaining informed consent from participants

Every young person will be provided with a participant information sheet when invited to take part in the study, or after making contact with the research team. Parents/carers will receive their own information sheet. These will provide information about the possible benefits and risks of taking part and participants will be given the opportunity to discuss the study with their GP or member of the research team before providing consent. When contacted by potential participants, the researcher will discuss participation and ensure eligibility. Those under 16 years will require parental consent. Those 16 years and over will be encouraged to seek parent/carer involvement and, if positive, a parent/carer will also be consented. Consent will be taken from young people and their parent/carer (if applicable) either in written form or verbally through a recorded telephone conversation with a member of the research team. Only after consent has been provided and the baseline measures completed will randomisation occur. Each participant will be given a £10 gift voucher (sent via email) as a token of gratitude for their time and participation following completion of 13, 26 and 52-week follow-up questionnaires. The explicit wishes of the participants will be respected, including the right to withdraw from the study at any time without giving a reason. During informed consent, participants will be advised that the data they provide up until the point of withdrawal will be used. Contact details for further information will be provided.

#### Safeguarding

YP Face IT is a self-help programme designed for use without additional input from health professionals. However, our research and the British Psychological Society's recommendations for online interventions^51^ have informed a safeguarding/supervision plan. Online data provided by young people will be reviewed online and ‘signed off’ each week by a researcher and a nurse/GP from their practice (if trained), who will check diary entries and qualitative data for safeguarding concerns (eg, signs of abuse or self-harm). Any concerns will be recorded as an adverse event, reported to the chief investigator and discussed with our clinical psychologist advisor who, if necessary, will make contact with the young person or parent/carer. Any further concerns or actions will be reported to the participant's GP who will respond following their usual protocols. This follows advice from our experts and replicates the established model of supervision by trained ‘administrators’ in primary care used by the ‘Beating the Blues’ online intervention. We will record compliance and compare whether researchers and practice staff identify the same entries as cause for concern. Email enquiries from participants will be sent to a single address managed by researchers (Monday–Friday, 9:00–17:00) who will record details and time taken to respond. Our acceptability studies found young people rarely use this service but consider it important to know professionals are available.

Young people specifically requested an online forum within YP Face IT; these are known to offer beneficial social support.^52^ ^53^ Users must agree to forum ‘rules’ imitating those used by ChildLine and health-related website forums. The forum will be moderated Monday–Friday. If young people ‘report abuse’, posts are automatically removed and the research team are notified immediately via email. No such problems arose in our acceptability studies. We will record time spent moderating the forum, participants' usage and discuss experiences of using this facility during interviews. This will inform future intervention costing and whether the forum is retained for any future RCT.

#### Data protection/confidentiality

All data will be stored in accordance with the Data Protection Act, 1998. Participant identifiable information will be locked in a filing cabinet within a secure office accessible only to the researchers. All participants will be allocated an ID to anonymise their data and identifiable data will be stored separately to anonymised data. Data entered by participants (eg, diary entries) are saved (protected by VeriSign security) on the website database, which is only accessible to the researchers, and the web designers who comply with University and NHS data protection policies and who will provide IT support for this and any subsequent studies. Practice staff named as supervisors for YP Face IT will only have access to the data of those participants who belong to their practice.

#### Research governance, safety and the conduct of the study

The study procedure and intervention were assessed to be of low risk for patient safety and it was decided an independent data monitoring committee was not required and interim analyses will not be conducted. A trial steering group will be set up with an independent chair and at least two other independent members and will meet 6 monthly. It will provide overall supervision of the study on behalf of the funders and the sponsor and will concentrate on the study progress, protocol adherence, patient/participant safety, consideration of new information and ensure that the study is conducted to the standards set out in Guidelines for Good Clinical Practice.^54^ The senior management team (principle investigator, researchers, clinical psychologist, health economist and statistician) will meet monthly to discuss protocol adherence, any protocol amendments for ethical approval by NRES Committee South West and study progress. Only members of the study management group will have access to the final data set.

#### Dissemination of research findings

The outcome of this feasibility study will inform our plans for a definitive trial. In order to maximise the success and take-up of such a study, and the use of the intervention if it is shown to be beneficial, we will ensure that this feasibility study is appropriately disseminated. Our advisory group will provide advice on the dissemination of the findings to patients, parents/carers and the participants themselves. Throughout the study, we will produce newsletters for participants to update them on our progress. The main outcomes and findings of this feasibility study will be disseminated via publications in a range of peer-reviewed journals. We will attend local primary care events to raise awareness of the study, the YP Face IT intervention and our findings among health professionals. Additional plans to provide feedback to the participating GP practices and charities supporting those with conditions affecting appearance will be developed with stakeholders during the study.

## Discussion

### Limitations and strengths of the YP face IT feasibility study

The study aims to establish whether it is acceptable and feasible to conduct an RCT to evaluate YP Face IT as an adjunct to TAU in a primary care setting. The data collected during the study will be essential to inform the design and delivery of a large-scale definitive trial. The need to improve support for young people distressed by appearance-altering conditions has been recognised by parents and NHS health professionals for some time.^15^ Strengths of this study include the development of an innovative, easily accessible online intervention with the potential to improve outcomes for young people struggling with appearance-related concerns, a population who currently have limited access to evidence-based psychosocial support. However, there are some limitations to the study. First, allocation to TAU may be confounded if young people and their parents are disappointed and try to seek help elsewhere, however, this will be dependent on available alternative resources, which are currently limited. To account for this, we will record TAU received by participants via the health economic data collection tools, note reviews and participant interviews. Second, we require participants to have access to the internet and this might restrict access to those with lower socioeconomic status and is something to consider for the future RCT. Finally, the self-report measures selected might not be sensitive to all aspects of improvements and may result in reporting bias as young people will not be blinded to their allocation. Despite these limitations, if an RCT is considered feasible, in the future, we hope that YP Face IT will be made widely available throughout the NHS to support those young people with visible differences who are struggling with appearance-related distress.
