# Supplementary material for: Study protocol of the YP Face IT feasibility study: comparing an online psychosocial intervention versus treatment as usual for adolescents distressed by appearance-altering conditions/injuries
Source: BMJ Open. 2016 Oct 3;6(10):e012423. doi: 10.1136/bmjopen-2016-012423 (PMC5073580; doi:10.1136/bmjopen-2016-012423)
Supplement: supplementary appendix [file bmjopen-2016-012423supp_appendix1.pdf]

Centre for Appearance Research
Faculty of Health & Applied Sciences
University of the West of England
Bristol, BS16 1QY Tel: 0117 3283513

YP Face IT: supporting young people with conditions or injuries that affect their appearance

CONSENT FORM for young people

Please tick box if you agree

1. I have read the **Information for Young People** (Version 2 dated 30/4/14) ☐
2. I understand it and I have been able to ask questions. ☐
3. I know that it's OK to stop taking part in the study if I want to, without saying why. ☐
4. I know that all the information I give is private and anonymous (no one will know it's me) and it will only be used for research. ☐
5. I know I will need to fill in questionnaires, I might be interviewed and this interview will be recorded, and I might be asked to do YP Face IT. ☐
6. I know that you will tell my GP that I am taking part in this study and that you may check my GP records at the end of the study. ☐
7. I know that if you are worried about my safety during the study you may need to talk to me, and maybe my parents or my GP. ☐
8. To make sure the study is being done properly, I understand that information about me in my doctors notes and from this study may be looked at by people who inspect research. I agree that these people can look at this information. ☐
9. I agree to take part in this study ☐

______________________ ______________ ____________________
Young person's name Date Signature

If you are under 16 years old please make sure you have talked about this study with a parent/carer. You will also need to get their permission and they will need to sign below.

______________________ ______________ ____________________
Parent or carer's name Date Signature

The following part will be completed by the researcher when you return your form:

Researcher	Date	Signature
