# Supplementary figures and images for: Study protocol of the YP Face IT feasibility study: comparing an online psychosocial intervention versus treatment as usual for adolescents distressed by appearance-altering conditions/injuries
Source: BMJ Open. 2016 Oct 3;6(10):e012423. doi: 10.1136/bmjopen-2016-012423 (PMC5073580; doi:10.1136/bmjopen-2016-012423)

## Appendix 2: Flow chart of the feasibility trial process

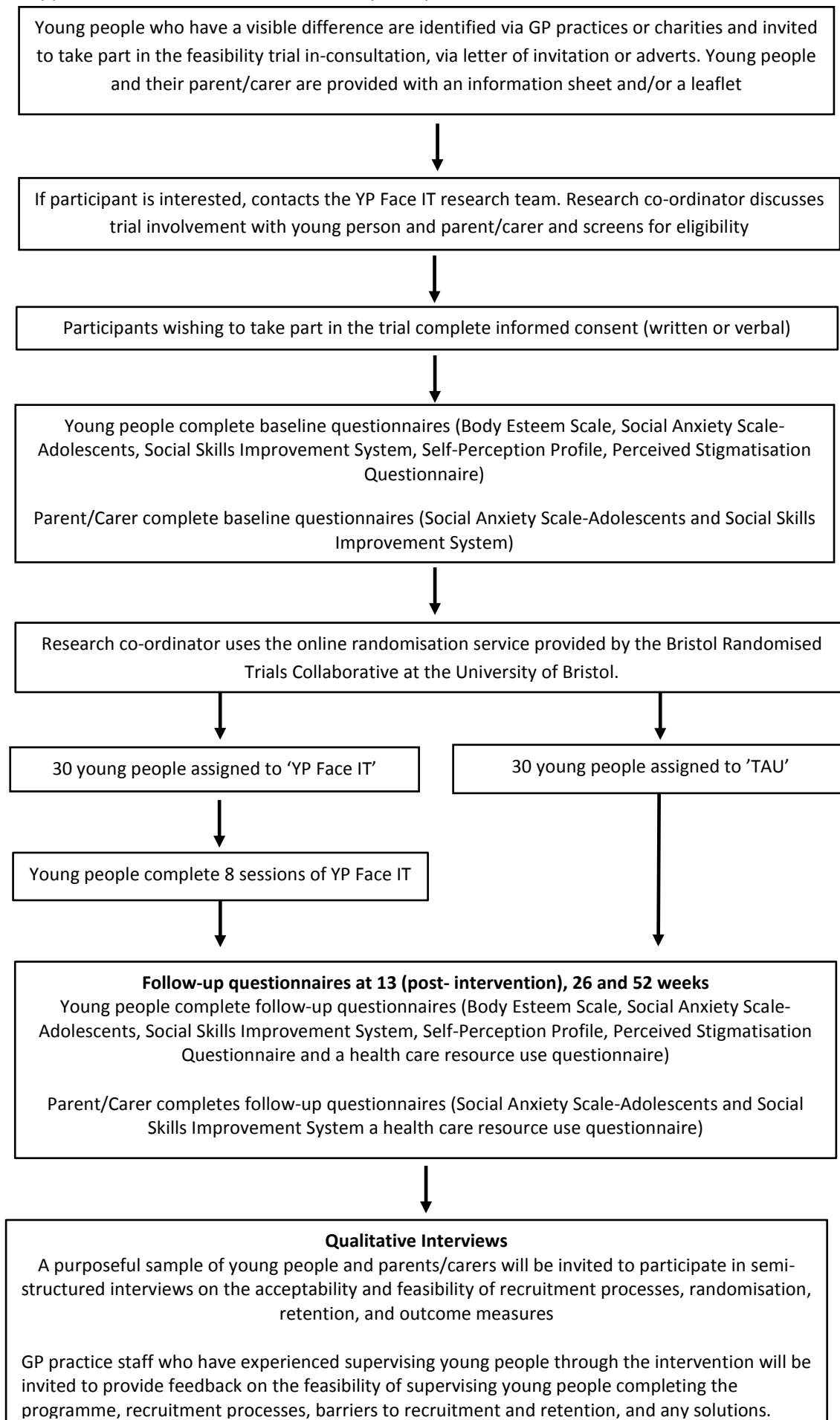

Supplement: supplementary appendix [file bmjopen-2016-012423supp_appendix2.pdf]
