# Supplementary material for: Study protocol of the YP Face IT feasibility study: comparing an online psychosocial intervention versus treatment as usual for adolescents distressed by appearance-altering conditions/injuries
Source: BMJ Open. 2016 Oct 3;6(10):e012423. doi: 10.1136/bmjopen-2016-012423 (PMC5073580; doi:10.1136/bmjopen-2016-012423)
Supplement: supplementary appendix [file bmjopen-2016-012423supp_appendix3.pdf]

Interview schedule at week 13: for young people

Questions for all young participants

- We are interested to find out about how good our recruitment methods were.
  - How did you hear about the study?
  - What did you think about the posters, leaflets and information sheets we gave you.
    - Can we improve them in any way?
    - If so how?
  - If you were asked to join the study by your doctor or nurse what did you think about the way you were asked?
  - If you saw an online advert what did you think of it?
  - Can you think of any way we can improve the way we recruit young people?

- We would like to know more about what you think of the way we allocated you to either the YP Face IT group or control group (the group that did not get YP Face IT.
  - Which group are you in?
  - How do you feel about being in that group?
  - Have there been any problems because you are in that group?
  - Can you think of any way we can improve this experience for young people?

- We would like to know what you think of the questionnaires we asked you to fill in.
  - Did you complete the questionnaires?
  - If not, why?
  - If you did,
    - how long did it take?
    - did you have any problems?
    - can we improve them in any way?

Questions for Control group only

- Can you tell us if you've had any support or information about your appearance worries since being part of the study?
  - If so, can you tell me about it?
  - What did you think of the support or advice?
    - Was it helpful?
    - If so, why
    - If not, how could it be improved?

Questions for Intervention group only

- We would like your views on using YP Face IT please.
  - What did you think of working through YP Face It?
      - What was good about doing it? What was not so good? What was difficult?
  - Did you complete all the sessions
    - If not can you tell me which… and why those and not others?
    - How can we encourage YPs to complete YP Face IT?

- How useful was the advice?
  - What in particular?
    - Have you used any skills?
- Did you complete the final session 8 quiz?
  - If not can you tell me why?
  - If you did what did you think of it?

- How does the programme reflect your own experiences of having a visible difference?
  - Can you identify with it?
  - Were there any difficult situations that you've had that were not covered in the programme?
- What did you think of the tasks and activities within YP Face It?
  - Did you feel encouraged to take part in the activities?
  - Were they easy, or difficult? In what way?
  - Can they be improved?
- What did you think of the 'set reminder' system?
  - Did you receive any reminders to complete the programme?
    - What did you think of the reminders
    - What else can we do to encourage or remind YPs to complete the programme and homework activities?
- What did you think of the discussion forum?
  - Did you use it?
    - If so, how?
    - How could it be improved, if at all?
- What did you think of the journal?
  - Did you use it?
    - If so, how?
    - How could it be improved, if at all?
- Did you need to use the e-mail service?
  - If so what for?
  - Can that service be improved?
  - Do you think the service is important or needed?
- Did your parents or carers or friends help you with YP Face It at all?
  - If so can you tell me how?
- What did you think of the guidelines we provided parents so that they can help you with YP face IT?
  - Can they be improved? If so how.
- Is there anything else you would like to say about YP Face IT?

.

Extra question:

- Have you ever talked to your doctor about worries you have around your appearance?
- If yes, what was your experience?
- If no, why did you not approach your GP?

- Having been in this study, would you be more likely to speak to your GP about any appearance worries
